# Epidemiological investigation of dengue fever outbreak and its socioeconomic determinants in Banadir region, Somalia

**DOI:** 10.1186/s12879-024-09276-2

**Published:** 2024-04-11

**Authors:** Mohamed Abdelrahman Mohamed, Nuralein Yusuf Hassan, Marian Muse Osman, Saido Gedi, Bisma Abdullahi Ali Maalin, Kasim Mahdi Sultan, Bashiru Garba, Ali Abdirahman Osman, Abdinasir Yusuf Osman, Abdifatah Diriye Ahmed

**Affiliations:** 1 National Institute of Health, Ministry of Health, Mogadishu, Somalia; 2 Ministry of Health and Human Service, Mogadishu, Somalia; 3 Africa Field Epidemiology Network, Mogadishu, Somalia; 4 Department of Public Health, Faculty of Medicine, and Health Science, SIMAD University Mogadishu, Mogadishu, Somalia; 5 Department of Veterinary Public Health and Preventive Medicine, Faculty of Veterinary Medicine, Usmanu Danfodiyo University, Sokoto, Nigeria; 6 Faculty of Veterinary Medicine and Animal Husbandry, Somali National University, Mogadishu, Somalia

**Keywords:** Dengue fever, Risk factors, Banadir region, Somalia, Outbreak

## Abstract

**Background:**

Dengue has become an alarming global problem and is endemic in many countries, particularly in tropical and subtropical countries. The aim of this study was to investigate dengue fever outbreak in Banadir Region, Somalia, to understand the risk factors (time, place, personal characteristics).

**Methods:**

A descriptive cross-sectional study was undertaken to determine the levels of circulating anti-dengue virus antibodies and DENV NS1 antigen among Banadir Region residents, while a questionnaire survey was conducted to understand the clinical and demographic characteristics of the patients.

**Results:**

A total of 735 febrile patients were studied, with 55.6% men and 44.3% women. The majority of the participants were children aged 14 years and younger. Among them, 10.8% tested positive for IgM antibodies against dengue virus (DENV), while the prevalence of DENV NS1 antigen was 11.8%. Fever and myalgia were the most common symptoms observed in the DENV-positive patients.

**Conclusions:**

A dengue fever outbreak has been confirmed in Banadir region, Somalia. This study provides information on the most affected districts and identifies risk factors contributing to DF outbreaks. The study recommends improving outbreak readiness and response, particularly in surveillance and laboratory diagnostics, by fostering intersectoral collaboration and establishing regulatory frameworks for financial and operational participation.

## Background

Dengue fever (DF) is an infectious disease spread by mosquitos caused by the dengue virus (DENV), a member of the Flavivirus genus and the Flaviviridae family [1]. DF is common in warm, tropical climates, causes flu-like illness, and spreads to humans through the bite of an infected female *Aedes aegypti* mosquito. Unlike other mosquitoes, *A. egypti* is a daytime feeder; its peak biting periods are early in the morning and the evening before dusk. The *Aedes aegypti* mosquito lives close to human populations, laying its eggs in water-filled containers inside the house or surrounding areas, including unused bottles, containers, discarded waste, and tires that hold water [2].

In more than 50% of cases, DENV infection is asymptomatic, but it can also mimic other endemic fever diseases in Africa (such as malaria and chikungunya fever) [3, 4].

Patients infected with DENV may present with a broad range of clinical manifestations, such as dengue fever (DF), severe dengue (SD), dengue hemorrhagic fever (DHF), and dengue shock syndrome (DSS).

Symptoms usually begin three to fourteen days after infection. These can include high fever, headache, vomiting, muscle and joint pain, and a characteristic skin rash (maculopapular rash) [5]. There is no effective antiviral treatment for dengue, but recovery usually takes two to seven days. The best way to protect against the dengue virus is to avoid mosquito bites. Importantly, these mosquitoes are also vectors of chikungunya, yellow fever, and Zika viruses. Infection can also be transmitted by blood transfusion or organ transplantation and can be transmitted vertically from mother to child. The mosquito becomes infected when it bites a person whose blood contains the dengue virus [6]. Hence, early diagnosis and management of symptoms are essential to reduce the risk of complications and prevent the further spread of the virus.

According to the WHO, the incidence of dengue has grown dramatically around the world in recent decades, with global warming and urbanization thought to be two of the main causes of this rise. The condition was first described in 1779, and in the early 20th century, the viral etiology and method of transmission were identified. Dengue has become a global problem since the Second World War and is endemic in many countries [7]. Over 50% of the world’s population in tropical and subtropical countries is at risk. Approximately 3.6 billion people are currently at risk of dengue infection in more than 100 countries in Asia, America, and Africa [8]. Before the year 2000, DENV-2 was the most commonly recorded serotype in Africa. It was the cause of multiple epidemics in East Africa (Somalia, Djibouti, Kenya, and Tanzania) as well as a sylvatic emergence with sporadic human cases in West Africa (Senegal, Burkina Faso). Only Mozambique (1985) and Somalia (1993) saw the emergence of DENV-3 [9]. Dengue fever infections in Africa remain largely unknown, but recent outbreaks suggest that key areas of the continent may be at increased risk of dengue transmission, and an estimated 390 million dengue infections occur worldwide annually. Somalia also suffered an outbreak of dengue fever in 2011 with the death of three peacekeepers from the African Union Mission in Somalia (AMISOM) in Mogadishu as a result of the infection [10]. A majority (82%) were positive for dengue virus (DENV). The second outbreak of dengue fever was reported in 2013 when 60 cases of dengue fever were detected in the country. Dengue fever remains high in Somalia because of the practice of storing water in open containers, which provides breeding grounds for the vector that transmits the disease.

On 18th October 2022, the Office of Emergency Department of the Federal Ministry of Health was notified of a death case of dengue and two confirmed cases in Banadir region, Somalia. The first case of dengue fever was confirmed in the Banadir Region on 11th September 2022. Active case outbreak investigation and contact tracing started in all districts of the Banadir region in October, and the investigation continues to date. On October 22, 2022, a Rapid Response Team (RRT) was established with members of the Federal Ministry of Health, the Public Health Emergency Department (FMOH), and the National Institutes of Health (NIH) with the sole aim of investigating the suspected dengue outbreak and proposing an appropriate response to contain the outbreak in the Banadir region, Somalia, covering a period between Oct 19th, 2022, and Jan 4th, 2023. To advance the readiness and response to the outbreak, particularly at community and policy-making levels, further description and information related to the issue were needed, particularly because of the absence of any previous updated assessment studies, therefore to close the gap we aimed to confirm the occurrence of the dengue outbreak, to describe the dengue cases by time, place, and personal characteristics, and to propose recommendations and improvements for future prevention of dengue fever.

## Method

### Study setting

**Fig. 1:**
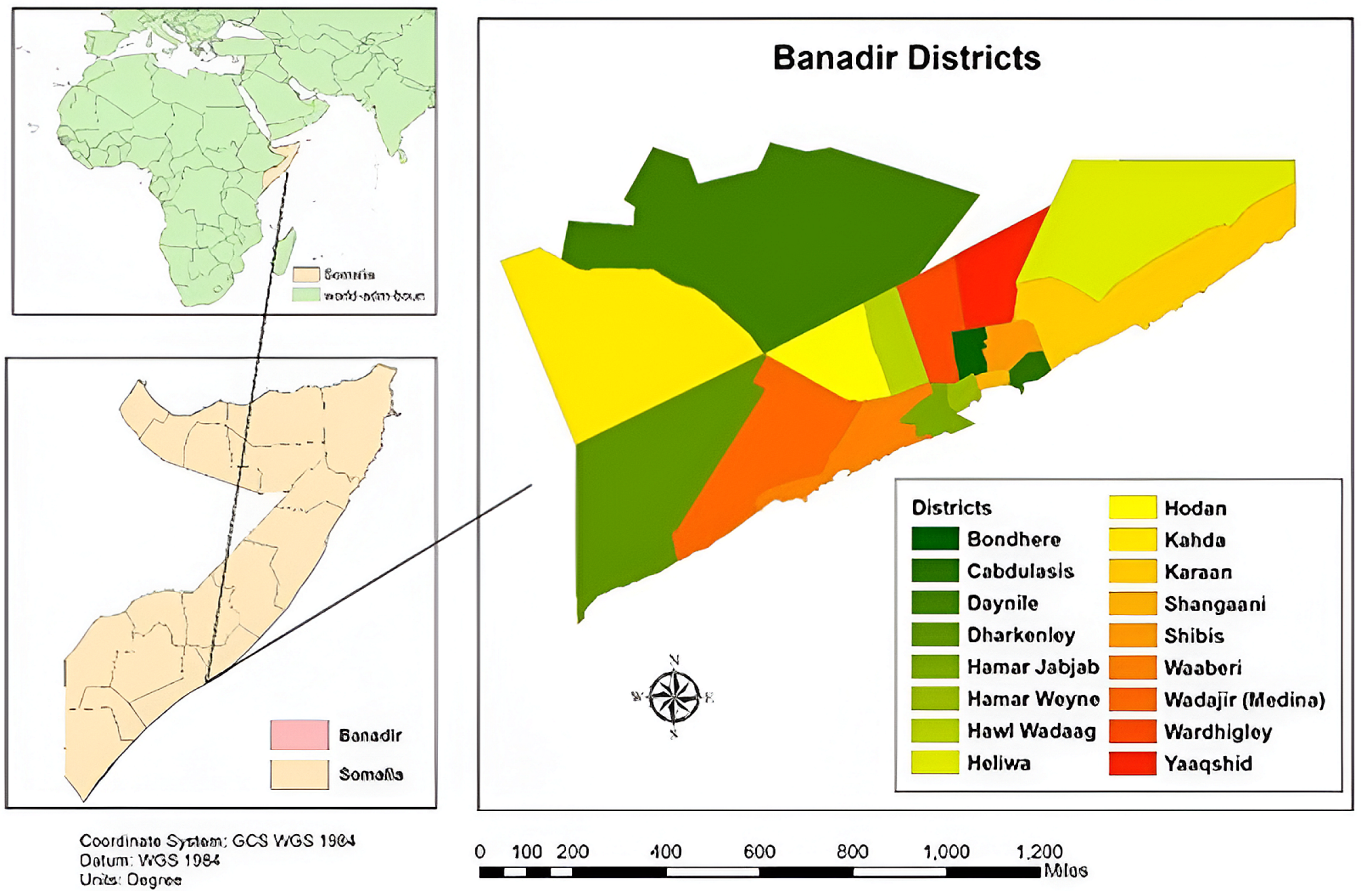
Graphical representation of the study location and map of the various districts within the Banadir region, Somalia

This investigation of a suspected dengue fever outbreak was conducted from November 20, 2022, to January 4, 2023. The study was conducted in Banadir Region, which consists of 17 districts: Wadajir, Dharkeynley, Daynile, Warta Nabada, Howlwadag, Waberi, Hamar Jajab, Hamar Wayne, Bondhere, Karan, Yaqshid, Hiliwa, Kahda, Hodan, Shibis, Abdiaziz, and Shangani (Fig. 1). The Banadir Region is bordered to the northwest by the Lower and Middle Shabelle regions and to the southeast by the Indian Ocean. Although it is by far the smallest administrative region in Somalia, it has the largest population, estimated at 2,647,351 (PESS 2022).

### Study design

A descriptive cross-sectional multicentric facility-based study approach was employed in this study to determine the seroprevalence of dengue virus infection among Banadir region residents, while a standard questionnaire was administered to understand the clinical and demographic characteristics of the patients. Descriptive epidemiological analysis was used to describe the outbreak using person, place, and time variables.

### Case definition

The WHO standard case definition of dengue fever was used to enroll the cases into the following categories.

#### Suspected dengue fever

Defined as any resident person in the Banadir region who has developed a sudden fever of more than 38 °C for 2 to 10 days with at least two of the following signs and symptoms: myalgia, maculopapular rash, hemorrhagic manifestations, or leukopenia between Sep 2022 and January 2023.

#### Confirmed dengue fever

Any resident of the Banadir region with a suspected case (Fever, myalgia, rash, hemorrhagic manifestations, or leukopenia.) with laboratory confirmation of IgM positivity for DENV.

### Inclusion criteria

All patients with oral temperature ≥ 38 °C, fever with at least one specific symptom (headache, joint pain, myalgia, maculopapular rashes, hemorrhagic manifestations), and residing in the study area regardless of age, gender, ethnicity, or tribe were included.

### Exclusion criteria

All Patients who lived outside the Banadir region and those that have been diagnosed with dengue fever were excluded.

### Data collection procedure

Data were collected by reviewing existing medical records and creating a line list with suspected and epidemiologically linked cases at the health facilities as probable cases. A WHO line list standardized tool designed to capture, essential data, for investigating and monitoring disease outbreaks questionnaire with variables such as demographic characteristics and an Arboviral line list was used to collect the information on the cases (https://www.who.int/emergencies/outbreak-toolkit/standardized-data-collection-tools). Face-to-face interviews were conducted with residents of the Banadir region at their homes, and where patients who met the profile of “suspected” were found, blood samples were aseptically collected. The collected blood sample was transported to the national laboratory for confirmation.

### Laboratory investigation

A serum sample was separated from the whole blood collected from suspected patients by centrifugation at 1500 × g for 4 min and tested for DENV NS1 antigens and the presence of circulating anti-DENV immunoglobulins in the Public Health Laboratory, National Institute of Health, Somalia. The Standard Q Arbo Panel I (Z/D/C/Y) test is a rapid multiplex immunochromatographic lateral flow assay for the detection of IgM to Dengue virus and the Dengue NS1 antigen. The sensitivity/specificity of the SD biosensor anti-DENV IgM is 71.8%/83.5%, while the DENV nonstructural protein 1 (NS1) glycoprotein has a sensitivity and specificity of 90.0%/90.2%, respectively. According to the manufacturer, 10 µL of serum sample was added, followed by the addition of 3 drops of assay diluent buffer. A positive NS1 test result confirms dengue virus infection without providing serotype information. However, a negative NS1 test result does not rule out DENV infection; hence, cases with negative NS1 were tested for the presence of dengue IgM antibodies to determine possible recent dengue exposure.

### Data analysis procedure

All data generated were collated, cleaned, and analyzed using IBM® SPSS® Statistics version 26 statistical software (USA). Descriptive statistics were used to express the results of place and personal information and presented as a table of frequency and percentage. An epicurve was used to show dengue fever cases by date of onset. The chi-square test was used to compare categorical variables. A p-value of < 0.05 was considered significant.

## Results

The demographic characteristics of the participants in all the districts of the Banadir region studied are presented in Table 1. A total of 735 febrile patients were recruited for the study, with 55.6% (409) being men and 44.3% (326) were women, with a male: female ratio of 1.3:1 (Table 1). The febrile participants were predominantly children, with 72.3% of them aged 14 years and below (*p* = 0.15) and the majority 24.9% (183) coming from Hodan district, followed by Wadajir.

**Table 1:** Demographic characteristics of participants based on IgM serological assay

Variables		+/*n*	95% CI:	*P*-value
Gender	Male	52/357	14.6 (11.1–18.7)	0.00
	Female	27/299	9.0 (6.0–12.9)	
Age	<1	21/139	15.2 (9.7–22.3)	0.15
	1–4	19/157	11.5 (6.9–17.5)	
	5–14	26/171	14.5 (9.6–20.1)	
	15–64	13/155	7.7 (4.1–13.1)	
	> 64	0/34	0	
District	Daynile	19/90	21.1 (13.2–30.1)	0.00
	Dharkeynley	15/83	8.2 (4.7–13.2)	
	Hamar jajab	0/15	0	
	Hiliwa	2/12	16.7 (2.1–48.4)	
	Hodan	13/170	7.6 (4.1–12.7)	
	Howlwadag	3/40	7.5 (1.6–20.4)	
	Kahda	5/28	17.9 (6.1–36.7)	
	Karan	3/15	20 (4.3–48.1)	
	Wadajir	13/132	9.8 (5.3–16.3)	
	Warta nabada	3/21	14.3 (3.0–36.3)	
	Yaqshid	2/32	6.3 (0.8–20.8)	
	Others*	1/18	5.8 (0.1–28.7)	

* Others refers to districts with less than 5 admitted patients

The simultaneous IgM antibody detection to identify patients with early infection and the detection of the DENV NS1 antigen results of individual patients revealed that a total of 79 of the febrile patients had circulating IgM antibodies against DENV, with an overall prevalence of 10.8%. Of this number, 52 were male, and 27 were female. Each of the DENV-positive patients was correlated with the clinical manifestations, and the results showed that fever 100% (79) and myalgia 75.9% (60) were the most common symptoms, followed by joint pain 60.0% (45) and maculopapular rashes 36.7% (29) (Table 2). only 7.6% (6) cases were found to exhibit hemorrhagic manifestations indicative of severe dengue. Notably, all the DENV-positive cases were found to be significantly correlated with the major clinical signs of the disease (*p* = 0.00).

**Table 2:** Clinical signs of the IgM (*n* = 79)

Clinical signs	Positive		*P*-value
	Yes	No	
Fever	79 (100%)	0	0.00
Joint pain	45 (60.0%)	34 (40.0%)	0.00
Myalgia	60 (75.9%)	19 (24.1%)	0.00
Maculopapular Rashes	29 (36.7%)	50 (63.3%)	0.00
Hemorrhagic manifestations	6 (7.6%)	73 (92.4%)	0.00

Analysis of the NS1 antigen showed a prevalence of 11.8%, representing 87 febrile patients. This is slightly higher than the results of the DENV IgM antibody assay, which indicated that 10.8% (79) patients were positive (Table 3).

**Table 3:** Dengue NS1 antigen results from all participants

Variables		+/*n*	95% CI:	*P*-value
Gender	Male	58/351	16.4 (12.7–20.1)	0.00
	Female	29/297	9.7 (6.6–13.7)	
Age	< 1	22/138	15.9 (10.3–33.1)	0.42
	1–4	20/156	12.1 (7.4–18.3)	
	5–14	27/170	15.1 (10.1–21.4)	
	15–64	18/150	11.3 (6.7–17.4)	
	> 64	0/34	--	
District	Daynile	19/90	21.1 (13.2–30.9)	0.00
	Dharkeynley	16/82	19.3 (11.4–29.4)	
	Hamar jajab	0/15	--	
	Hiliwa	2/12	16.7 (2.1–48.4)	
	Hodan	15/168	8.9 (5.1–14.2)	
	Howlwadag	7/36	17.5 (7.3–32.8)	
	Kahda	4/29	14.3 (4.0–32.7)	
	Karan	4/14	26.7 (7.8–55.1)	
	Wadajir	13/132	9.2 (4.9–15.6)	
	Warta nabada	3/21	14.3 (3.0–36.3)	
	Yaqshid	2/32	6.2 (0.8–20.8)	
	Others*	2/17	11.7 (1.5–36.4)	

*Others: District with less than 5 admitted patients

Similarly, the correlation of the NS1-positive patients and the clinical signs also indicated fever at 100% (87), myalgia (*n* = 68, 78.2%), joint pain at 60.9% (53), maculopapular rashes at 34.5% (30), and hemorrhagic manifestation 6.9% (6) (Table 4).

**Table 4:** Clinical signs of the Dengue NS1 antigen (*n* = 87)

Clinical signs	Positive		*P*-value
	Yes	No	
Fever	87 (100%)	0	0.01
Joint pain	53 (60.9%)	34 (39.1%)	0.00
Myalgia	68 (78.2%)	19 (21.8%)	0.00
Maculopapular Rashes	30 (34.5%)	57 (65.5%)	0.00
Hemorrhagic manifestations	6 (6.9%)	81 (93.1%)	0.00

**Fig. 2:**
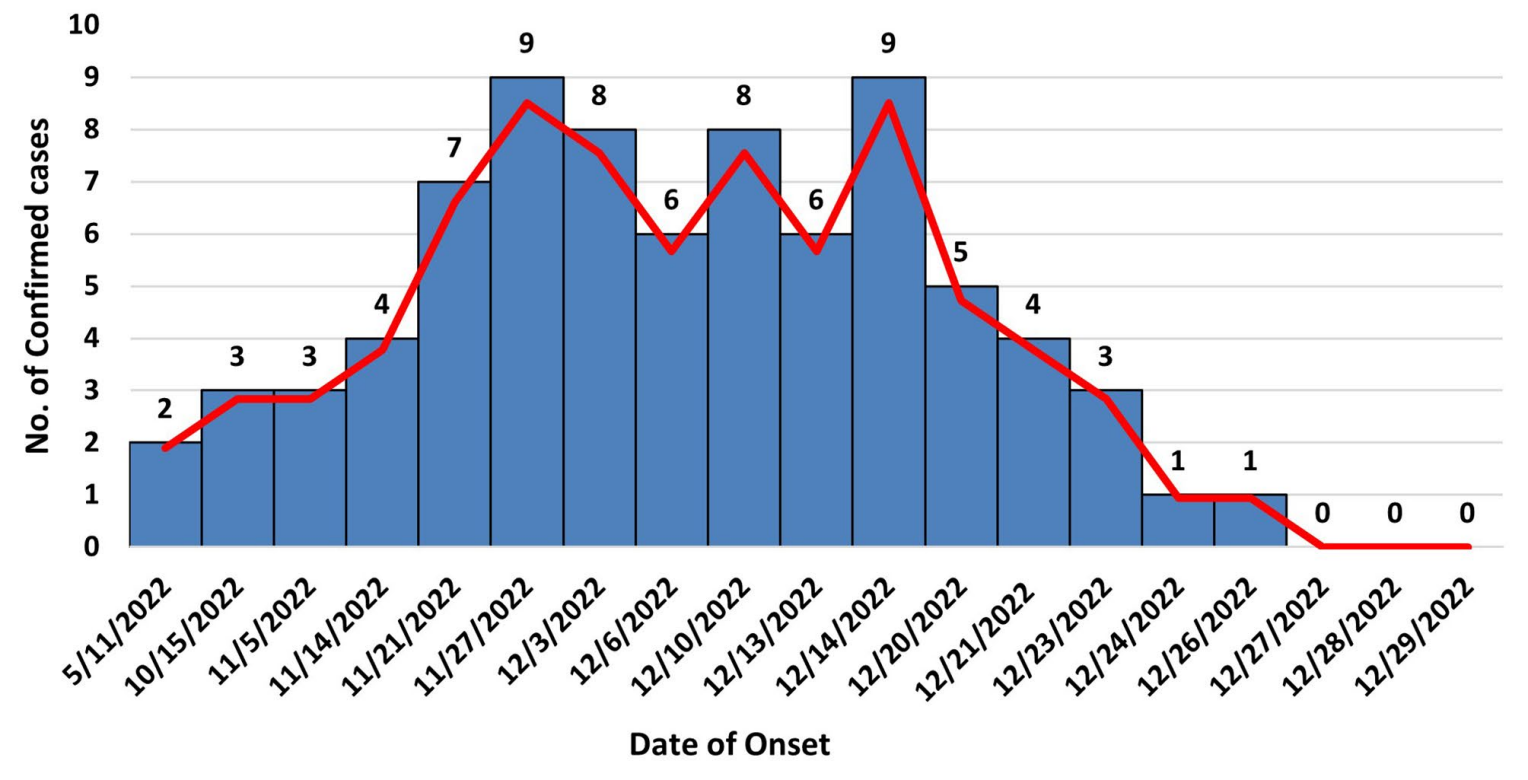
Epicurve of confirmed Dengue fever case by date of onset

The epi curve demonstrates that the confirmed cases of Dengue fever initially exhibited a low incidence, with two cases reported on November 5th and three cases reported on October 15th (Fig. 2). Subsequently, the number of cases steadily escalated, reaching a peak of nine cases on November 27th and again on December 14th, 2022. Following this peak, there was a notable decline in the number of cases, ultimately reaching zero new cases by January 5th, 2023.

## Discussion

The current study documented a total of 79 febrile patients with confirmed dengue fever across the 17 districts in the Banadir region of Somalia with 10 deaths across the province during the two-month data collection period, with Deynile being the hotspot reporting the highest dengue cases (21.1%) with no associated death. A dengue fever outbreak was recently reported in Somalia in October 2022, where a total of 211 suspected cases were reported in Banadir. Similar to the findings of this study, the most affected districts are Deynile, Hodan, and Wadajir [11]. Dengue is not a very common disease in Somalia; however, in recent years, there appears to be a rise in the states, affecting people from all walks of life, particularly those with a poor standard of living. Moreover, there is poor risk communication, awareness, and community engagement regarding the disease.

The result of the current investigation where more males are affected compared to females is in congruence with the results obtained by the WHO Somalia office as well as a hospital-based molecular survey conducted in two military hospitals in Mogadishu in 2011 [10, 11]. The significant disparity between males and females is suggestive of the existence of gender-related differences in dengue incidence, which can be attributed to job exposure differences as well as time away from home [12, 13]. On the other hand, the lower number of dengue incidences recorded in females may be because Somali women’s traditional clothing requires them to be covered fully according to the teachings of Islam, and in most cases, they are full-time housewives with minimum movement out of their houses, hence limiting their exposure to mosquito bites and subsequent dengue transmission [14, 15].

The higher incidence of dengue in children (> 1 to 14 years) in the current study is consistent with the general pattern of dengue fever. Although dengue affects all age groups, 90% of dengue occurs in children less than 18 years of age [16, 17] The childhood morbidity from infectious diseases and mortality rate in Somalia is among the highest in the world [18]. Statistics have shown that one out of seven Somali children dies before they turn five, and the majority of the causes are infectious diseases. Other potential contributors to the high rate of dengue cases observed among children could be the poor economic status of the parents. An estimated 70% of the population is reported to be living below the international poverty line, with the numbers projected to be higher in rural areas and members of the internally displaced population residing in the capital city.

In this study, fever was present in 100% of the patients in both the IgM antibody tests and the dengue NS1 antigen tests. This is followed by myalgia, joint pain, and maculopapular rashes. Dengue fever is classified based on clinical manifestations, with patients having fever, nausea, vomiting, rash, myalgias, arthralgias, rash, or leukopenia described as having mild dengue [19]. Based on the signs and symptoms observed in this study, it is believed that the majority of the patients in this study had mild dengue, except for a small percentage (6%) that showed hemorrhagic signs.

Serological detection of DENV in human blood is an effective method of predicting impending epidemics. The current study observed 10.8% DENV positivity in blood based on the IgM assay to detect early infection. However, analysis of the dengue NS1 antigen in blood revealed a slightly higher prevalence of 11.8%. The detection of DENV IgM together with NS1 antigen in the serum is used for routine differentiation between primary and secondary infections, which can be useful in monitoring the acute phase of dengue infections. While the NS1 antigen is found from Day 1 and up to Day 7 after the onset of fever, IgM can be detected on Day 4 to 5 of illness, while combined testing with an NS1 antigen and IgM antibody can usually provide a diagnostic result that is comparable with molecular screening.

### Study limitations

We recognize some limitations to this study, such as the inability to run serotype analysis of DENV and investigate the possibility of coinfection, the limited geographical study area to the Banadir region, the inability to identify the mosquitoes and larval breeding sites in the field beyond visual inspection using entomological investigations, and the underreporting of cases due to health workers diagnosing dengue fever as malaria disease.

## Conclusion

We observed 11.8% confirmed dengue cases among the febrile patients, with the majority being male. It also appears that poor hygiene and overcrowding could be a significant risk factor for dengue fever, as demonstrated by the highest number of cases coming from the Deyniile and Hodan districts, which are inhabited by the majority of the IDP population in the Banadir region. Our findings will provide additional data-based evidence that the Ministry of Health, policymakers, and other stakeholders in the healthcare service delivery system in the country can use to develop guidelines aimed at the prevention and control of the disease in the country.

## Data Availability

All data are available from the corresponding author.
